# Targets and Combs: A Case of Lupus Enteritis

**DOI:** 10.7759/cureus.7892

**Published:** 2020-04-29

**Authors:** Mehdi Faraji, Edgar Gutierrez, Alex Glotser, Jugal Thaker, Christopher Gammarano

**Affiliations:** 1 Radiology, Louisiana State University Health Sciences Center, Shreveport, USA; 2 Radiology, Brookwood Baptist Medical Center, Birmingham, USA; 3 Internal Medicine, Louisiana State University Health Sciences Center Shreveport, Shreveport, USA; 4 Radiology, Mayo Clinic, Jacksonville, USA

**Keywords:** lupus, enteritis, target sign, comb sign

## Abstract

Lupus is a common autoimmune disorder with the potential to affect all organ systems. Lupus enteritis is a rare complication that is seen in a subset of patients that present with gastrointestinal symptoms. Its diagnosis commonly involves imaging, showing bowel wall edema as the target sign and vascular engorgement of bowel vessels as the comb sign on CT scans. These findings can help guide the diagnosis, but they are nonspecific and are also found in other conditions that cause bowel wall ischemia. These symptoms are reversible if treated with immunosuppressants. Unfortunately, recurrence is common in lupus enteritis and perforation needs to be ruled out on presentation. In this report, we present the case of a patient with known lupus who was diagnosed with lupus enteritis on imaging and was treated with immunosuppressants. The patient's symptoms resolved subsequently. Our case highlights the fact that the appropriate diagnosis and management of this condition require physical exams, labs, and imaging.

## Introduction

Lupus is a well-documented autoimmune disorder with multi-organ involvement that can be diagnosed via its many autoantibodies. Common entities affected are skin, muscle, heart, blood, and the central nervous system. Though rare, it can involve the gastrointestinal system, and enteritis is one such entity. Lupus enteritis is defined by the British Isles Lupus Assessment Group (BILAG) as vasculitis or inflammation of the small bowel, usually diagnosed with imaging and/or biopsy findings. It most commonly presents as abdominal pain. Other signs and symptoms include ascites, nausea, vomiting, diarrhea, and fever. This entity is fatal in 11% of active cases and thus requires expeditious diagnosis. If left untreated, this can lead to bowel infarction and perforation. The ileum and the jejunum are also commonly involved. CT scan will show bowel wall edema in 98% of cases, and 71% of cases will show target sign and/or combs sign. The target sign is due to bowel wall edema and combs sign represents mesenteric vessel engorgement [1]. This edema is reversible, as seen on repeat CT imaging of many cases after an acute lupus enteritis event [2]. These signs are helpful, but can also be seen in other situations that cause or mimic intestinal ischemia. Endoscopy is not helpful for diagnosis in many cases. Immunosuppressive agents can resolve lupus enteritis, but recurrence is common. If perforation occurs, surgical intervention is required emergently [3]. It is believed that lupus enteritis is caused by immune complex deposits and complement activation. Complement activation can then cause microvascular injury and increased permeability leading to submucosal edema of the intestine [4]. Lupus enteritis is rarely diagnosed on biopsy, with rates as low as 6% [5].

## Case presentation

A 28-year-old African-American male with a past medical history of systemic lupus erythematosus (SLE), hypertension, peptic ulcer disease, and end-stage renal disease (ESRD) secondary to lupus nephritis presented with a one-day history of severe abdominal pain. The abdominal pain was diffuse and sharp; it was associated with nausea and he had experienced two episodes of non-bloody, non-bilious vomiting prior to arrival. He also reported multiple watery bowel movements. The patient denied fever, chills, sick contacts, or recent travel history. The patient reported similar episodes on and off for a week prior to arrival.

The patient had an extensive workup, which showed low complement C4 levels, while erythrocyte sedimentation rate and C-reactive protein were elevated. His anti-Sjögren’s-syndrome-related antigen A (anti-SSA), anti-Sjögren’s-syndrome-related antigen B (anti-SSB), anti-RNP, anti-double-stranded DNA, and anti-Smith were also positive. The patient was started on Plaquenil (Sanofi S.A, Paris, France) 200 mg daily.

**Figure 1:**
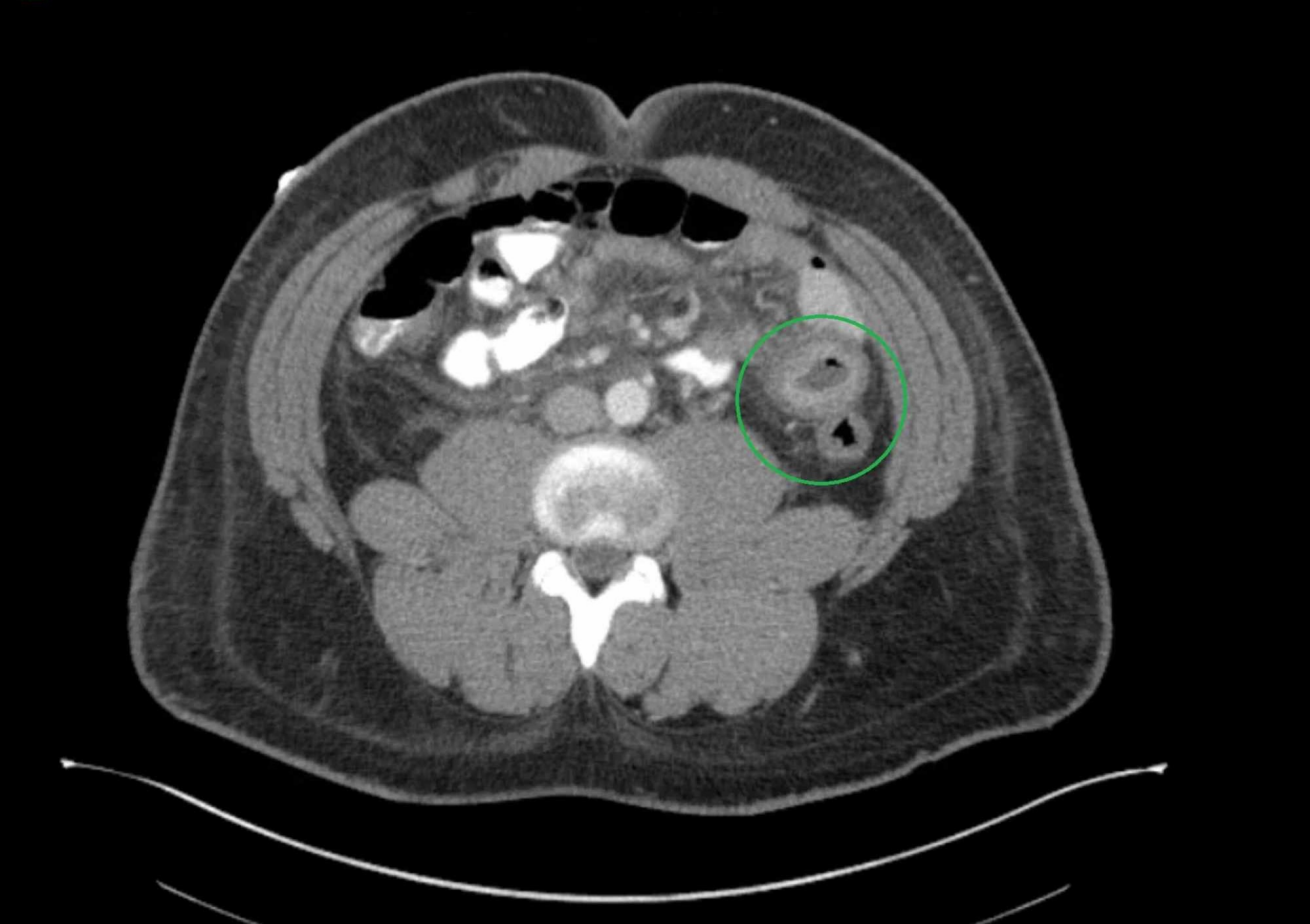
Target sign on CT of the abdomen (green circle) CT: computed tomography

**Figure 2:**
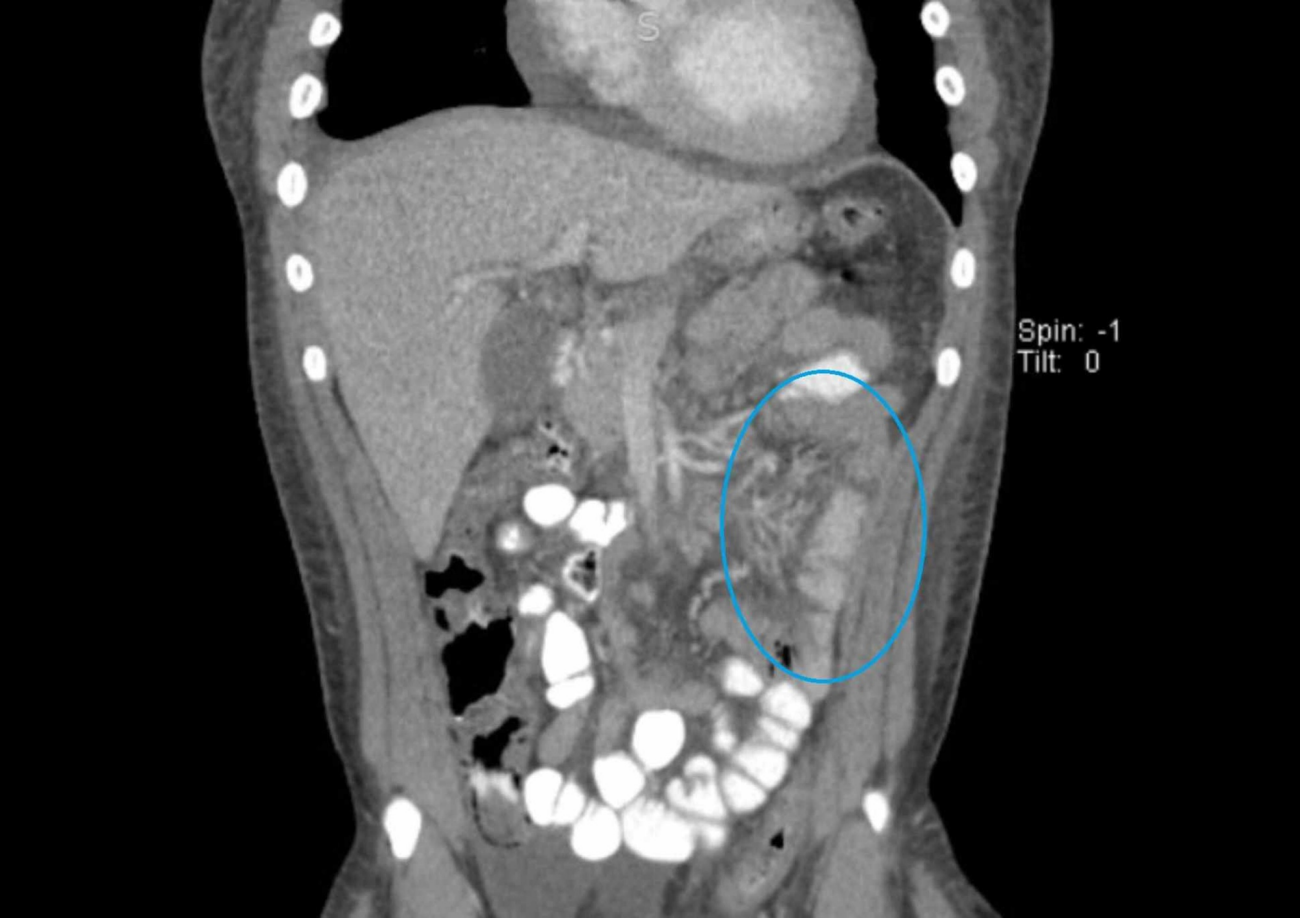
Combs sign on CT of the abdomen (blue oval) CT: computed tomography

His pain was treated with morphine intravenously as needed. His other medications included hydralazine 25 mg, lisinopril 40 mg, and nifedipine extended-release 60 mg for blood pressure control. CT scan of the abdomen was consistent with lupus enteritis, which included target sign in the small bowel and comb sign of the vasculature to the bowel (Figures 1, 2).

Gastroenterology performed an esophagogastroduodenoscopy and colonoscopy, revealing normal-appearing colon; he tested negative for gastritis, duodenitis, and *Helicobacter pylori* on gastric biopsy. The patient was discharged home on a regimen including a proton pump inhibitor, Carafate (Allergan, Dublin, Ireland), and Plaquenil.

## Discussion

Lupus is a well-known disease that is diagnosed with a myriad of autoantibodies and has the potential to affect any and all organ systems. If a patient with lupus has gastrointestinal symptoms, lupus enteritis should be on the differential, even though it is a rare complication of lupus. Diagnosis of lupus enteritis is very challenging and imaging can be very helpful. Lupus enteritis is defined by BILAG as small bowel vasculitis or inflammation diagnosed with imaging and/or biopsy. It can be fatal and requires expeditious diagnosis and treatment [1].

We presented a case of a patient with known lupus who presented with abdominal pain, nausea, vomiting, and diarrhea and was subsequently diagnosed with lupus enteritis. Imaging studies showed classical features of lupus enteritis, which included the target sign, which is due to bowel wall edema. The enhancing layers of the bowel wall constitute the outer layer, which is composed of muscularis propria and serosa and the inner mucosa layer, while the hypoenhancing layer is the middle edematous submucosal layer, which gives us the target sign [6]. The combs sign was also detected on CT imaging, which is due to mesenteric vessel engorgement. Our patient was treated with Plaquenil with a resolution of his symptoms. Though surgical intervention was not necessary in our case, we should be aware that these patients will likely have a recurrence, and perforation is not unheard of.

## Conclusions

Lupus enteritis is a rare subset of lupus complications that can be dangerous and requires expeditious diagnosis and treatment. Recurrence is common and perforation can also occur. The imaging findings on CT scan of the abdomen is the gold standard of diagnosis, though these same findings can be found in other situations that can cause or mimic intestinal ischemia. We presented a case of lupus enteritis with a resolution of symptoms. Appropriate management of this condition requires a collaborative effort involving clinical exams, labs, and imaging.
